# Positioning berries in nutritional psychiatry: potential for prevention and co-therapy in mental health

**DOI:** 10.3389/fnbeh.2025.1622242

**Published:** 2025-09-23

**Authors:** Rafael Fernández-Demeneghi, Marilú Domínguez-Pantoja, Alma Gabriela Martínez-Moreno, Isidro Vargas-Moreno, Rodrigo Ramirez-Rodriguez

**Affiliations:** ^1^Instituto de Investigaciones en Comportamiento Alimentario y Nutrición, Universidad de Guadalajara, Ciudad Guzmán, Mexico; ^2^Facultad de Medicina, Universidad Veracruzana, Campus Xalapa, Xalapa, Mexico; ^3^Instituto Politécnico Nacional, Mexico City, Mexico

**Keywords:** nutritional psychiatry, mental health, berries, functional food, neuroplasticity

## Introduction

Mental health is a critical component of wellbeing and a growing global concern. The World Health Organization (WHO) defines health as complete physical, cognitive, and social wellbeing, emphasizing that mental health is not merely the absence of mental disorders but a foundation for individual and societal functioning (WHO, 2021). Psychiatric conditions—including mood disorders, anxiety, and other psychiatric conditions—are multifactorial in origin and increasingly prevalent worldwide.

While pharmacological treatment remains the first-line therapy for psychiatric disorders, behavioral interventions such as psychotherapy have gained prominence, paving the way for complementary approaches to mental wellbeing (Gilbert, 2020). Lifestyle-based strategies—particularly dietary interventions—are gaining recognition. This perspective has led to the emergence of “*nutritional psychiatry.”* This field investigates how nutrition influences brain function and emotional health and seeks to elucidate the biological mechanisms through which dietary components impact mental health outcomes. Key physiological targets include oxidative stress regulation, neuroinflammation, neurotransmission, synaptic plasticity, and the gut–microbiome–brain axis, interconnected systems highly responsive to nutritional modulation (Logan and Jacka, 2014).

Within this context, berries (e.g., strawberries, raspberries, blueberries, blackberries, and others) have garnered attention due to their rich polyphenols, vitamins, minerals, fiber, and bioactive compounds with neuroprotective potential. This opinion article explores the emerging role of berries as functional foods in psychiatric nutrition, highlighting their potential to complement traditional interventions in preventing and managing mental health disorders. Nevertheless, clinical research focused on psychiatric populations remains scarce, particularly in trials involving individuals with diagnosed conditions, underscoring the need for targeted and well-designed studies to understand their therapeutic relevance better.

## Berries: a functional food

Berry fruits—such as blueberries, blackberries, strawberries, raspberries, and blackcurrants—are rich in essential nutrients, including vitamins, minerals, fiber, and diverse bioactive compounds (Seeram, 2012). Their primary phytochemicals are phenolic compounds, notably flavonoids (e.g., anthocyanins, flavonols, flavones, flavanols, flavanones, isoflavonoids), tannins, and phenolic acids (Golovinskaia and Wang, 2021). Certain berry constituents have been linked to reduced mortality and lower risk of cancer, cardiovascular and metabolic diseases, as well as improved cognitive function, neuroinflammation, glucoregulation, cerebrovascular health, neurotransmission, and hippocampal neurogenesis—mechanisms relevant to mental health (Aguilera, 2024).

While berries share common phytochemical categories, their specific profiles—including the type and concentration of bioactive compounds—can differ substantially across species and cultivars. These compositional distinctions may underlie unique biological effects, adding nuance to their potential applications in mental health. As such, future research would benefit from a more differentiated approach that considers berry-specific characteristics when assessing their relevance in psychiatric contexts.

**Figure 1:**
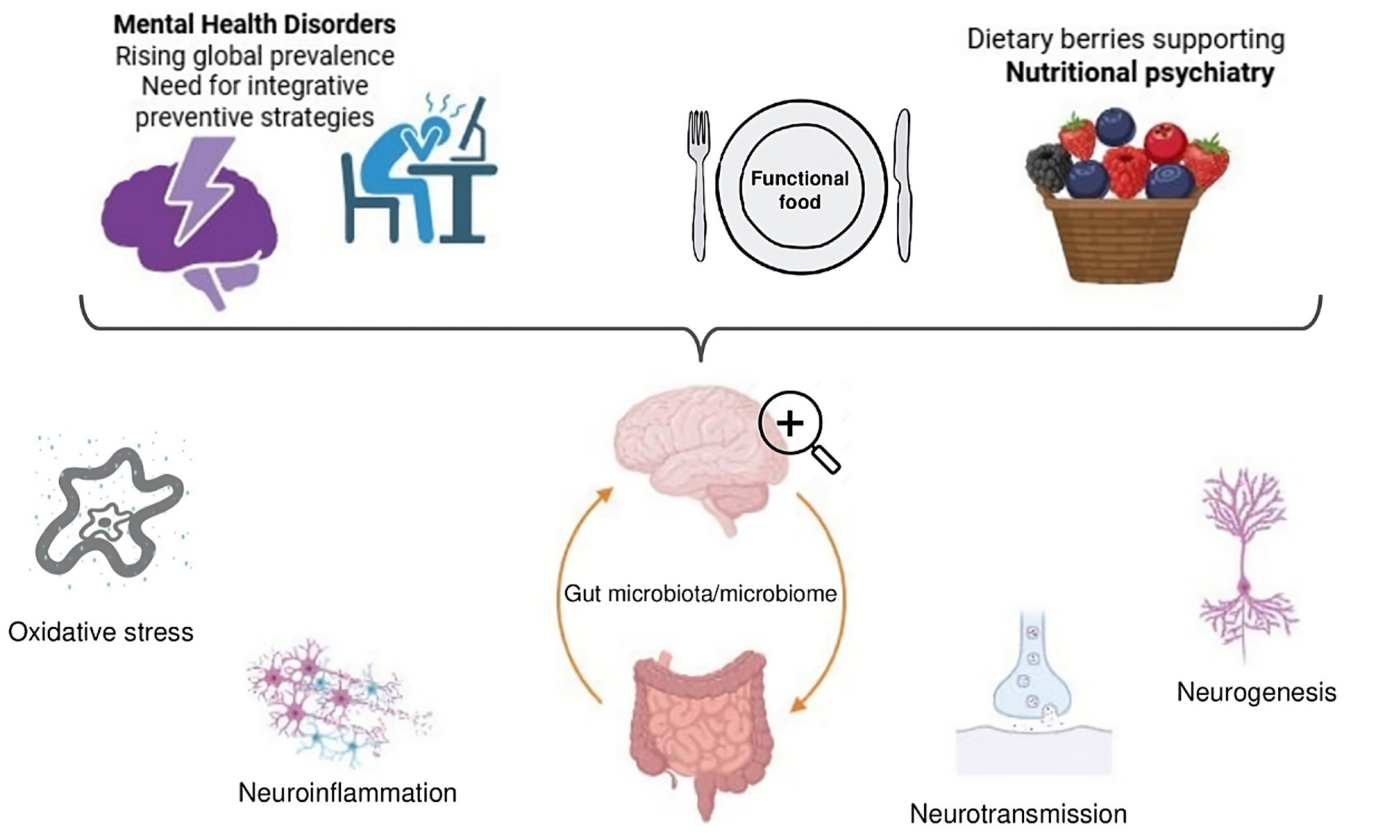
Integrative framework linking berries, nutritional psychiatry, and mental health mechanisms.

Psychiatric disorders involve dysregulation in dopaminergic, serotonergic, and glutamatergic pathways, alongside alterations in neurotrophic factors, immune and neuroendocrine systems, and epigenetic mechanisms (Sutkowy et al., 2021; Fang et al., 2020). This piece aims to discuss key findings on the potential mechanisms by which berry-derived compounds may influence mental health outcomes (Figure 1).

## Oxidative stress

Oxidative stress results from an imbalance between reactive oxygen/nitrogen species (ROS/RNS) production and the body's ability to neutralize them (Cecerska-Heryć et al., 2022). The brain is especially vulnerable due to its high oxygen demand, abundance of peroxidizable lipids, and limited antioxidant defenses (Zalachoras et al., 2020). Excess ROS impairs synaptic plasticity, neurogenesis, and contributes to neuronal degradation (Salim, 2017; Singh et al., 2019). To counteract oxidative damage, cells rely on enzymatic antioxidants—superoxide dismutase (SOD), catalase (CAT), glutathione peroxidase (GPx), and glutathione reductase (GR)—and non-enzymatic antioxidants like glutathione (GSH), vitamins A, C, E, and trace elements such as zinc (Salim, 2017; Niedzielska et al., 2016; Salim, 2014).

Although pharmacological strategies exist, antioxidants continue to be explored as adjunctive options for their neuroprotective potential in psychiatric disorders. Berries contain up to four times more antioxidants than other fruits, mainly due to phenolic compounds and vitamin C (Golovinskaia and Wang, 2021). In psychiatric populations, elevated oxidative stress markers and reduced antioxidant defenses are consistently reported, supporting the rationale for antioxidant-based interventions (Grabnar et al., 2011; Rasmus and Kozłowska, 2023).

For example, supplementation with 30 g of freeze-dried black raspberries for 4 weeks enhanced CAT and GPx activity in healthy male smokers, mitigating oxidative stress related to cigarette exposure (Handelman et al., 1996). While informative, such findings do not establish therapeutic relevance in psychiatric populations. Observational studies indicate that individuals with depression often consume fewer antioxidant-rich foods and exhibit lower plasma concentrations of vitamins C and E (Payne et al., 2012; Huang et al., 2019). Vitamin C supplementation has shown antidepressant effects and mood enhancement, although such results are not specific to berry intake. In postmenopausal women, antioxidant-rich diets have correlated with reductions in anxiety and oxidative stress markers (Wu et al., 2022), yet the distinct contribution of berries remains to be clarified.

In summary, although berries exhibit strong antioxidant potential, more robust clinical trials are needed to determine whether this biochemical activity translates into meaningful mental health outcomes in individuals with diagnosed psychiatric conditions.

## Inflammation

Phytochemicals in berries exhibit anti-inflammatory effects by modulating immune cell activity and inhibiting pro-inflammatory mediators such as TNF-α, IL-1β, IL-6, IL-8, and C-reactive protein (CRP; Pap et al., 2021). For instance, strawberry extract reduces IL-8 secretion and downregulates TNF-α, IL-1β, and iNOS via MAPK pathway inhibition (Fumagalli et al., 2016; Lee et al., 2014). In a short-term clinical study, a 7-day intake of a strawberry beverage attenuated postprandial increases in IL-6 and CRP in overweight adults following a high-fat, high-carbohydrate meal (Edirisinghe et al., 2011). Although these results are promising, they were obtained in healthy individuals and not in clinical psychiatric samples.

Chronic low-grade inflammation, including neuroinflammation, is implicated in various psychiatric and neurodegenerative disorders (van Zonneveld et al., 2024). Depression, in particular, is strongly linked to immune-inflammatory mechanisms, with elevated levels of CCL2, CXCL10, proinflammatory cytokines, and CRP reported in affected individuals (Müller, 2013; Hong et al., 2016). Through MAPK and NF-κB pathway modulation, polyphenols demonstrate anti-inflammatory effects in preclinical and clinical models of depression (Wium-Andersen et al., 2014; Winiarska-Mieczan et al., 2023; Behl et al., 2022). Moreover, remission from depression has been associated with the normalization of inflammatory biomarkers, suggesting a possible mechanistic link between inflammation and symptom improvement (Wang et al., 2022).

Inflammatory mediators also induce indoleamine 2,3-dioxygenase, promoting tryptophan degradation to kynurenine and thus reducing serotonin availability—an essential factor in depression (Mondanelli et al., 2020). In animal models, blueberries reduced proinflammatory cytokines and anxiety in PTSD (Ebenezer et al., 2016), while blackberry extract decreased IL-6 and increased IL-10 in a bipolar disorder model (Chaves et al., 2020). While mechanistic evidence is encouraging, clinical research in psychiatric populations remains limited. Given the diversity in berry phytochemistry, future trials should consider species-specific effects and diagnostic relevance.

## Gut microbiota/microbiome

The gut microbiome plays a critical role in the gut–brain axis, influencing neurotransmission, inflammation, and blood–brain barrier integrity (Ebenezer et al., 2016; Chaves et al., 2020). Antioxidants may modulate gut microbiota by altering the redox environment, enhancing short-chain fatty acid (SCFA) production, reducing inflammation, and promoting beneficial bacterial growth (Silva et al., 2020; Majumdar et al., 2023).

The gut microbiota predominantly comprises Firmicutes and Bacteroidetes (~90%), with smaller proportions of *Proteobacteria, Actinobacteria, Fusobacteria*, and *Verrucomicrobia* (Pap et al., 2021; Neri-Numa et al., 2020). Diets rich in polyphenols and fiber improve microbial diversity and abundance of beneficial taxa. In contrast, Western-style diets can reduce species like *Faecalibacterium prausnitzii, Akkermansia muciniphila, Lactobacillus*, and *Bifidobacterium* (Neri-Numa et al., 2020; Healey et al., 2017), while polyphenol-rich diets improve microbial balance. Dysbiosis—microbial imbalance—has been linked to metabolic, immune, and neuropsychiatric disorders (Neri-Numa et al., 2020). For instance, antibiotic-induced dysbiosis increases depression risk by 20–50% (Lurie et al., 2015), with reduced Firmicutes levels noted in depressed patients (Bosch et al., 2022). Individuals with depression show lower levels of anti-inflammatory butyrate-producing bacteria (*Faecalibacterium, Coprococcus*) and higher levels of pro-inflammatory species (*Eggerthella*; Winiarska-Mieczan et al., 2023; Nikolova et al., 2021).

Altered microbiota also impairs neurotransmitter synthesis, including serotonin and dopamine, potentially contributing to psychiatric symptoms such as anxiety, depression, and schizophrenia (Horn et al., 2022). Specific taxa such as *Oscillibacter* and members of *Actinobacteria* and *Bacteroidetes* have been associated with depression (Naseribafrouei et al., 2014), while bipolar disorder is linked to increased *Bacteroidetes, Clostridiales*, and decreased *Faecalibacterium* (Evans et al., 2017; Rong et al., 2019). Anxiety correlates with reduced Lactobacillus and increased *Lachnospiraceae* (Ouabbou et al., 2020; Hemmings et al., 2017).

While research on berries is still emerging in this domain, some preclinical studies suggest they may modulate gut microbiota in ways relevant to mental health. For example, *Lycium barbarum* (goji berry) intake increased butyrate-producing bacteria and the expression of butyryl-CoA transferase, a key enzyme in SCFA synthesis, in mice (Kang et al., 2018). Both animal and human studies report reduced SCFA levels in depression (Deng et al., 2019; Szczesniak et al., 2016; Kelly et al., 2017), and butyrate has been shown to reverse behavioral deficits in animal models (Burokas et al., 2017). Given the anti-inflammatory and neuroactive properties of SCFAs, their depletion may contribute to mood disorder pathophysiology (Silva et al., 2020).

Although mechanistic links between berry fruits and gut microbiota modulation are promising, their effects in clinical psychiatric populations remain underexplored. Future research is warranted to clarify species-specific contributions and evaluate whether microbiome changes can support meaningful improvements in mental health.

## Neuroplasticity and behavior

Although the neurobiological basis of anxiety and depression remains partially understood, monoaminergic neurotransmitters—serotonin (5-HT), dopamine (DA), and norepinephrine (NE)—have long been central to their pathophysiology (Shao and Zhu, 2020; Olivier and Olivier, 2020). More recently, impaired neuroplasticity has emerged as a unifying mechanism across psychiatric disorders, with chronic stress inducing structural and functional changes in emotional brain circuits such as the prefrontal cortex, hippocampus, amygdala, striatum, and raphe nuclei (Drevets, 2004).

Within this context, growing attention has turned to the role of diet, particularly berries, as a source of bioactive compounds capable of modulating mood-related pathways (Fernández-Demeneghi et al., 2019b). Rich in polyphenols and anthocyanins, berries exert polypharmacological effects. Preclinical studies show these compounds inhibit monoamine oxidase A (MAO-A), increasing central levels of 5-HT, DA, and NE and enhancing receptor activity (Imran et al., 2021; Tomić et al., 2016; Dreiseitel et al., 2009). Additionally, certain flavonoids appear to interact with GABA_A_ receptors, suggesting an anxiolytic mechanism (Imran et al., 2021; Gutierres et al., 2014).

These biochemical actions align with observed behavioral and neuroplastic outcomes. Previous studies reported anxiolytic-like effects of blackberry extract in rats (Fernández-Demeneghi et al., 2019a), and wild blueberry anthocyanins reversed stress-induced dopaminergic and oxidative changes in the prefrontal cortex (Rahman et al., 2008). Similarly, anthocyanins reduced behavioral despair in rodents, comparable to the antidepressant mianserin, and improved neuronal integrity in the hippocampal CA3 region under oxidative stress (Drenska et al., 2008; Varadinova et al., 2013).

Berry extracts have also shown antidepressant-like effects in models of post-stroke depression. Extracts from *Hypericum androsaemum* and *Aristotelia chilensis* reduced immobility in standard behavioral tests (Nabavi et al., 2018; Di Lorenzo et al., 2019). While clinical evidence remains limited, some human studies are emerging. Blueberry juice has been associated with reduced depression risk in youth (Mestrom et al., 2024; Godos et al., 2018; Park et al., 2021), and anthocyanin-rich diets correlate with lower depressive symptoms in adults. A placebo-controlled trial further confirmed reduced depressive symptoms in adolescents after 4 weeks of blueberry supplementation (Fisk et al., 2020).

Beyond monoamines, berries also influence neuroplasticity. *Grewia asiatica* has shown pro-cognitive, anxiolytic, and antidepressant effects in rodents (Imran et al., 2021). Blueberry phytochemicals have been linked to increased neurogenesis in the dentate gyrus (Casadesus et al., 2004) and improved spatial memory alongside hippocampal remodeling (Rendeiro et al., 2012). These effects are believed to involve activation of ERK–CREB–BDNF and PI3K/Akt/mTOR pathways, leading to increased BDNF expression, synaptogenesis, and dendritic complexity (Vauzour et al., 2021; Fang et al., 2020).

Taken together, these preclinical findings suggest that berries may influence neurobiological pathways relevant to mood and cognition, although a significant gap remains in their clinical translation. Future studies should prioritize rigorous trials in psychiatric populations, using well-defined outcomes related to both symptoms and neurobiological markers.

## Discussion and future directions

Berries are increasingly recognized for their nutritional and neuroprotective properties, primarily due to their high content of polyphenols—particularly anthocyanins, vitamins, and other bioactive compounds. These constituents have demonstrated potential to modulate pathophysiological mechanisms central to mental health disorders, including oxidative stress, neuroinflammation, gut dysbiosis, and impaired neuroplasticity. However, while preclinical research robustly supports these effects, translation into clinical psychiatric populations remains limited.

Recent systematic reviews report that berry consumption can improve cognitive domains such as memory, executive function, processing speed, and attention (Bonyadi et al., 2022; De Amicis et al., 2022; Wang et al., 2023)—areas frequently compromised in depression, anxiety, and stress-related disorders. Observational data further support an inverse association between anthocyanin-rich fruit intake and depressive symptoms, perceived stress, and poor sleep (Micek et al., 2022), suggesting possible indirect or adjunctive benefits. However, much of the existing research relies on non-clinical samples. While biomarker improvements have been reported in healthy or cognitively impaired individuals, evidence of symptom reduction in well-characterized psychiatric populations remains scarce. To bridge this gap, rigorous trials are needed that define psychiatric diagnoses, assess symptom severity, and apply standardized measures across affective, cognitive, and neurobiological outcomes.

A further challenge is the heterogeneity of berry interventions. Phytochemical composition varies markedly by species, cultivar, ripeness, and processing. For instance, blueberries are rich in anthocyanins, while strawberries contain more ellagic acid and vitamin C (Miller et al., 2019). Additionally, the form of administration—whole fruit, juice, powder, or extract—significantly influences bioavailability and metabolic response (De Amicis et al., 2022; Wang et al., 2023). Future research should avoid overgeneralization and instead compare specific berry types, formulations, and polyphenol doses using standardized protocols.

Mechanistic evidence is also emerging. Aronia berry supplementation, for example, has been linked to improved arterial stiffness and greater gut microbial gene richness—particularly in butyrate-producing species—suggesting modulation of the gut–brain axis (Le Sayec et al., 2022). Although findings on oxidative stress are mixed, about one-third of biomarkers showed significant improvements in a recent review (Stote et al., 2023), pointing to plausible systemic effects even in the absence of direct psychiatric endpoints.

An essential but often overlooked issue is the potential for pharmacokinetic interactions. Some flavonoids can inhibit cytochrome P450 enzymes or modulate transporters like P-glycoprotein, potentially altering the metabolism of psychotropic medications. While such interactions are unlikely at dietary levels, the increasing use of concentrated extracts and nutraceuticals highlights the need for targeted pharmacological studies. Toxicological considerations, though rare, also merit attention. Certain berries or their unripe forms may contain saponins or solanine, compounds linked to gastrointestinal damage or possible carcinogenicity. Wild species like *Nandina domestica* are toxic to animals, though not typically consumed by humans (Fernández-Demeneghi et al., 2019b). As berry-based supplements become more common, further safety assessments and public health guidance will be essential.

Despite these limitations, berries offer a biologically plausible, low-risk, and multitarget intervention aligned with the principles of nutritional psychiatry. Their ability to modulate inflammation, oxidative stress, gut microbiota, and cognition makes them promising adjuncts to conventional care—particularly within lifestyle-based or personalized models.

In light of current evidence, several research priorities emerge: a) Mechanistic studies in humans to elucidate how specific berry compounds influence neurotransmission, synaptic plasticity, inflammatory cascades, and microbiota-derived metabolites linked to mood regulation; b) Clinical trials involving well-defined psychiatric populations, stratified by diagnosis, baseline symptomatology, and treatment status, using consistent and validated outcome measures; c) Comparative studies of different berry species, polyphenol doses, and delivery forms (e.g., whole fruit vs. extract vs. juice) to determine optimal therapeutic profiles; d) Longitudinal and preventive research in at-risk groups (e.g., adolescents, older adults, individuals exposed to chronic stress) to evaluate resilience-building effects and potential for primary prevention; and e) Safety and pharmacokinetic evaluations, particularly in populations using psychotropic medications, to guide clinical application.

Advancing toward a preventive and salutogenic model of mental health care requires more than symptom management—it calls for systemic strategies that foster emotional resilience and neurobiological integrity. Within this framework, nutrition is not a cure-all, but a meaningful, accessible, and underutilized tool. Including culturally relevant, locally available berries as part of dietary interventions offers a cost-effective and non-invasive approach to support mental health. As ‘Nutritional Psychiatry' evolves, integrating functional foods like berries may help bridge disciplines—linking neuroscience, nutrition, and clinical care in new and impactful ways.
